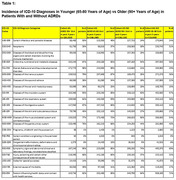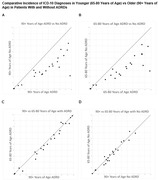# Comparative Comorbidity Profiles of Younger (65‐80 years of age) vs Older (90+ years) Individuals With or Without Alzheimer’s Disease and Related Dementias

**DOI:** 10.1002/alz.086117

**Published:** 2025-01-09

**Authors:** Stephen J. Peroutka

**Affiliations:** ^1^ PPD, Inc., Wilmington, NC USA

## Abstract

**Background:**

Alzheimer’s Disease and Related Dementias (ADRDs) patients have a well‐established increase in comorbid disorders compared to non‐cognitively impaired aged‐matched controls. Since ADRDs are considered age‐related disorders, it was hypothesized that younger ADRD patients should have a lower incidence of comorbid disorders than older ADRD patients.

**Method:**

Four patient cohorts were defined in the TriNetX Analytics Network database (comprised of anonymized healthcare records from >150 million patients who have received care at 80+ health care organizations in the United States). Cohort #1 was comprised of individuals, aged 90 and above, who had a healthcare visit within the past 3 years and who had received a diagnosis of AD (ICD‐10 Code G.30) or unspecified dementia (ICD‐10 Code F.02). Cohort #2 had the same criteria except that the patients were aged 65‐80 years. Cohorts #3 and #4 matched Cohorts #1 and #2, respectively, except that they had not received an ADRD diagnosis.

**Result:**

Diagnostic code data on the incidence of the 22 major subgroups in the ICD‐10 Coding system are provided in Table 1. As expected, the Cohort #1 90+ year old ADRD patients had a significantly higher incidence of comorbid disorders in nearly all ICD‐10 categories compared to Cohort #3 non‐ADRD 90+ year olds (shown graphically in Figure 1A). Similarly, Cohort #2 65‐80 year‐old ADRD patients had a significantly higher incidence of comorbid disorders compared to Cohort #4 non‐ADRD 65‐80 year‐olds (Figure 1B). However, despite the significant age difference between 65‐80 and 90+ year‐old cohorts, there were minimal comorbidity differences between the younger and older cohorts with ADRDs (Figure 1C) and without ADRDs (Figure 1D).

**Conclusion:**

The major finding of the present study is that the well‐documented increased incidence of comordid organ dysfunction in ADRDs appears similar in younger and older ADRD patients. These data indicate that a diagnosis of ADRD is associated with coincident multi‐organ dysfunction in a pattern that is nearly identical across ADRD age groups. These data suggest that the cognitive impairment associated with ADRDs is a single component of a multi‐organ senescense syndrome, characterized by symptoms of widespread and concurrent organ dysfunction.